# Assessment of Work Ability in People with Musculoskeletal Disorders in Primary Health Care: A Scoping Review of Self-Reported Tools

**DOI:** 10.1007/s10926-025-10323-1

**Published:** 2025-11-13

**Authors:** K. M. Tveten, A. Skaar, A. T. Onni, T. Dragesund, L. H. Magnussen, M. N. Tvedt, T. Ask

**Affiliations:** 1 Faculty of Health and Social Sciences. Department of Health and Functioning, Western Norway University of Applied Sciences, Bergen, Norway; 2 Faculty of Health and Social Sciences. Department of Welfare and Participation, Western Norway University of Applied Sciences, Bergen, Norway; 3 Department of Global Public Health and Primary Care, University of Bergen, Bergen, Norway; 4 Department of Addiction Medicine, Haukeland University Hospital, Bergen, Norway

**Keywords:** Assessment, ICF, Occupational health, Sickness absence, Tools, Self-report

## Abstract

**Purpose:**

Musculoskeletal disorders account for approximately 25% of consultations in primary health care, and sickness absence due to these disorders imposes a substantial economic burden on society. Early identification of workers at risk of sickness absence is crucial; however, there is no consensus on how to identify risk and guide work participation for individuals with musculoskeletal disorders. This scoping review aims to address the research question: Which tools (questionnaires or questions) are used to assess work ability and guide work participation in workers with musculoskeletal disorders, and how do they correspond to the codes in the International Classification of Functioning, Disability and Health (ICF) model?

**Methods:**

Through systematic searches on EMBASE, Medline, Pedro, CINAHL and AMED, we identified tools relevant to assessing work ability in primary health care for use in the early phase of sick leave. Items in the questionnaires were mapped into second level ICF codes.

**Results:**

The scoping review included 95 studies from an initial pool of 10,800 articles. The studies covered 25 tools, all except two of which were available in English, and ten were available in a Scandinavian language. The diverse evidence base for work ability assessment tools spans various ICF codes, with most questionnaires including items in the activity and participation domains. Few questionnaires addressed environmental and personal factors.

**Conclusion:**

The evidence base for work ability assessment tools is extensive, with significant variation in tools, linkage to ICF, and psychometric properties. This scoping review supports healthcare personnel selecting tools based on their purpose, time, body area, and dimensions covered, considering the duration of sickness absence and case complexity. Future research should incorporate environmental and personal factors for comprehensive evaluations.

**Supplementary Information:**

The online version contains supplementary material available at https://doi.org/10.1007/s10926-025-10323-1.

## Introduction

Sickness absence due to musculoskeletal disorders (MSDs) poses a significant challenge to the workforce worldwide, with far-reaching implications for individuals, employers, and society [1, 2]. According to the Global Burden of Disease (GBD), MSDs represent the largest cause of non-fatal health loss in Norway [3], accounting for approximately 33% of the sickness absence [4]. Labour and welfare policies underscore the workforce as a vital resource, aiming to enable as many individuals as possible to participate in the workforce [5]. This highlights the need for early identification of those at risk of prolonged sickness absence, allowing for timely interventions to support their ability to engage in meaningful work activities [6, 7].

Healthcare professionals, including general practitioners (GPs) and physiotherapists (PTs), play a critical role in managing patients with MSDs at risk of sickness absence or in the early phase of sickness absence. In a Scandinavian context, these workers are typically enrolled in primary health care, where the health care personnel engage with patients in the early stages of the disease process and includes preventive measures in addition to managing the disease. In this context, the concept of"work ability"is indispensable, as it provides a framework for assessment and intervention designed to foster continued work participation.

Work ability is a multifaceted concept influenced by individual factors, workplace elements, and societal contexts. In a systematic review by Lederer et al. [8], various definitions and conceptualisations of work ability were discussed, reflecting the diverse scientific, professional, and epistemological perspectives of the researchers involved. For this scoping review, the definition of work ability synthesises the contributions of Tengland [9], Ilmarinen [10], and Nordenfelt [11]. It is defined as “an individual’s capacity to perform specific tasks and roles required at work, considering their health, competencies, values, attitudes, and work environment".

A range of tools, both performance-based and self-reported, is available for evaluating work ability. Recently, two systematic reviews of tools for predicting a return to work (RTW) were published. The first, by Hurri et al. [12] was concentrated on performance-based tests. Performance-based tests and self-reported questionnaires measure different yet interrelated aspects of work ability, thus offering complementary information [13]. Self-reported assessments encompass aspects not addressed by performance-based tests, such as a person’s beliefs, motivation, and perceptions of health, functionality, and the work environment. These factors are crucial for obtaining a comprehensive view of work ability and guiding rehabilitation to promote work participation [14]. In the second review, Villotti et al.[15] examined specific items within tools that could cause work disability and predict RTW in people with MSDs and/or common mental disorders. Only predictive studies published in English or French were included, resulting in the identification of 68 different tools or questions with varying predictive value. Participants had differing lengths of sickness absence, and there were no restrictions on the time required to administer the tools.

Work ability and work function can sometimes seem similar and may appear in the same tools. Both constructs involve the interaction between an individual’s health and their work environment. A distinction is that while work function refers to actual performance in the workplace, work ability pertains to the potential to work [16]. Given the various conceptualisations of work ability [8], the assessment tools developed for this purpose may accentuate different aspects of the construct. These aspects can be contextualised using the International Classification of Functioning, Disability, and Health (ICF). The ICF provides a biopsychosocial framework encompassing the physical, mental, and social dimensions of functioning, environmental demands and personal characteristics that influence an individual’s capacity to meet these demands [17]. Although the ICF has not been designed explicitly to describe work (dis)ability or work functioning [18], it provides a framework that can also be used to determine functioning in the context of work [19]. In addition, the ICF can be used as a standard for comparison of assessment tools and provide in depth details on the content of the tool through coding items or questions to specific ICF codes.

Previous work has addressed tools for evaluating work ability, but certain gaps remain, requiring further research on some issues. A review by Sternberg et al. [20] was limited to studies that explicitly referenced the ICF and included only English- and German-language studies, thereby restricting the range of tools considered for measuring work functioning. Another systematic review by Gragnano et al. [21] had narrowed their scope to measures of individual psychosocial factors that could predict return to work (RTW) after MSDs and common mental disorders (CMD). However, neither review emphasised tools intended for use in the early phase of sickness absence, as recommended by health authorities [7].

### Purpose and Significance

The purpose of this scoping review is to give an overview of self-report tools suitable for use in primary health care settings during an early phase of sickness absence and map the items with codes from the ICF framework. The focus was on tools aimed at assessing work ability and guide work participation among employees with MSDs. A subsequent aim was to explore which tools were available in a Scandinavian language. The significance of this review is to offer valuable insights for clinicians to select the most appropriate tools in primary care settings.

## Methods

A protocol for the review was published at Open Science Framework (OSF), reg. https://doi.org/10.17605/OSF.IO/65CSA. The scoping review was carried out following 5 steps described by Khalil [22]. (1) identifying the research question; (2) literature search; (3) choosing relevant studies; (4) creating an overview of data; and (5) summarizing and identifying the significance for practice and possible further research.

### Stage 1: Identifying the Research Question

The topic for this review was identified through expert consultations with researchers within sickness absence, own experiences in clinical practice, and previous research indicating that physiotherapists require standardized tools to assess work ability[23, 24]. The formulation of the research question was inspired by the SPIDER framework [25]. The Sample of Interests were workers with MSDs. The Phenomenon of interest was work ability, specifically in a Scandinavian primary health care context. The Design of interest was studies presenting self-reported questionnaires or questions. The research type of interest was peer-reviewed papers in all languages. Through discussion, we arrived at the following research questions: Which tools (questionnaires or questions) are used to assess work ability and guide work participation in patients with MSDs? A sub-question was: Which tools are translated into a Scandinavian language? The term work ability was used in this paper as the person’s self-reported work ability.

### Stage 2: Literature Search

The systematic search of EMBASE, Medline, Pedro, CINAHL and AMED was carried out in June 2023 and updated in March 2025 by a scientific librarian (MNT). Additional sources and citation searches were made by checking reference lists and conducting citation searches on Google Scholar. In addition, Norwegian specialists were contacted and asked for knowledge on any other additional questionnaires.

### Stage 3: Choosing Relevant Studies

Identification of studies with relevant tools based on title and abstract was conducted independently by five authors (KMT, AS, ATO, TD, and TA). Further screening of the full text was done by two authors (KMT and TA). Conflicts were solved through discussion or resolved by a third author (AS). The PRISMA extension for Scoping Reviews (PRISMA-ScR) checklist [26] is available as a supplementary document.

We aimed to synthesise evidence that is most relevant to the practical application of work ability assessment tools in the context of primary health care for patients with MSDs. We decided to include tools used within the first eight weeks of sick leave, as the likelihood of returning to work decreases with longer absences [27, 28].

The screening was conducted against the following pre-determined inclusion criteria:

- Studies presenting self-reported questionnaires or sets of questions designed to assess and/or predict work ability and/or guide work participation in populations suffering from MSDs.
- Studies presenting tools suitable for use by healthcare personnel in primary healthcare settings. The administration of these questionnaires or questions should not exceed 15–20 min.
- Included studies encompassed generic tools used for a population with MSDs or tools specifically developed for patients with MSD.
- Studies involving samples with short-term sickness absence (≤ 8 weeks) were considered.
- Peer-reviewed publications of any publication year and language.

Studies were excluded based on the following criteria:

- tools designed for specific diagnoses (e.g., rheumatic or neurological diseases, cancer, specific MSDs such as hip and knee arthritis, frozen shoulder, or whiplash-associated disorders);
- tools for individuals on long-term sick leave (over 8 weeks) or receiving disability benefits and tools measuring physical function (e.g., Functional Capacity Evaluation); and
- studies on the translation of tools or questionnaires into non-Scandinavian languages were excluded.

### Stage 4: Create an Overview of Data

Data from the included studies were extracted with help from DistillerSR by one review author (TA) and verified by a second author (KMT). Disagreements were settled through conversation or, if required, with a third author (AS).

The data was charted for all articles using the following categories: name of tools, number of items, study population, measurement properties examined (reliability, validity, predictive ability, discriminant ability, responsiveness and normative data), time to administer in minutes, year of publication, and bodily area of concern. The time to administer in minutes was either extracted from statements in the article, or by the time taken to fill out by the review authors.

### Stage 5: Summary and Identification of Significance for Practice

The characteristics of each study were mapped, and the topics of each tool were presented according to the second level codes in the ICF framework. Each questionnaire was studied item by item, linking them to the ICF codes *b* for body function and structure,* d* for activity and participation, and *e* for environment. Items related to personal factors were noted and given a short description such as “sex”. For single items related to several codes, the range of codes was noted. As an example, item no. 5 in the Fear- Avoidance Beliefs Questionnaire “I cannot do physical activities which (might) make my pain worse…”, were linked to the codes d410-d460 related to mobility and pain was linked to b280. This process was conducted by one review author (TA) and verified by a second author (KMT). Disagreements were settled through conversation or, if required, with a third author (AS). Measurement properties of the included studies were described and presented in a table.

## Results

**Fig. 1:**
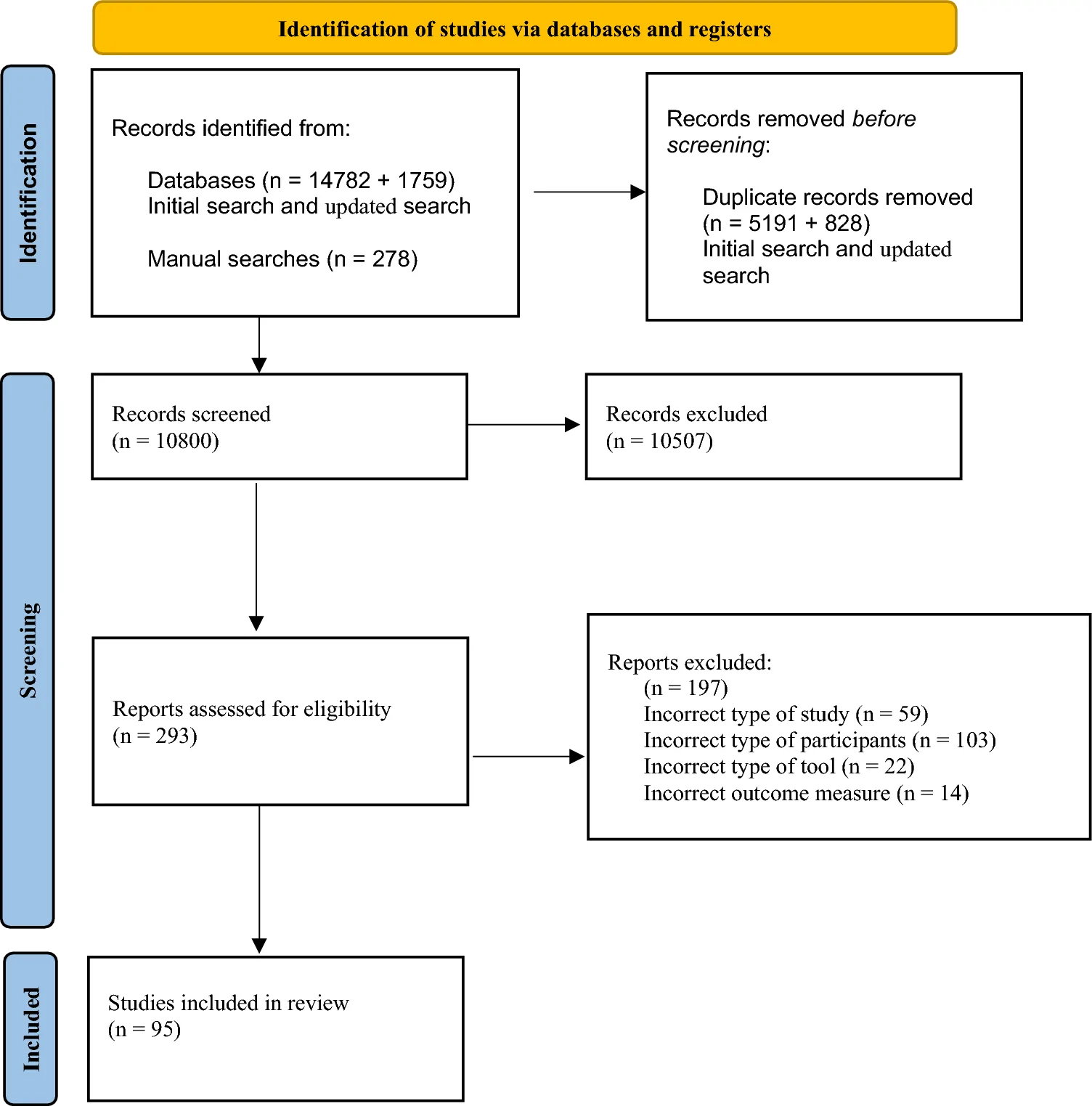
PRISMA flow diagram

The process of article selection is presented as a PRISMA diagram in Fig. 1. From an initial pool of 10,800 articles, we included 95 studies resulting in the identification of 25 distinct questionnaires for assessing work ability in individuals with MSDs. Some tools are variants of the same form (ALBPSQ and OMPQ – 25, Örebro Musculoskeletal Screening Questionnaire, OMSQ- 21). In these questionnaires, the items are similar, but tailored for slightly different target groups. Further, the Örebro also has shorter versions available (OMPQ-SF – 10, OMSQ – 12). Work Limitations Questionnaire (WLQ-25), (WLQ-16), and Work Role Functioning Questionnaire (WRFQ) are also related questionnaires. Additionally, the Return-To-Work Self-Efficacy scale RTWSE-10 is a short version of RTWSE-19.

### Study Characteristics

The identified questionnaires are detailed in Table 1, (supplementary file), summarising the main characteristics of each instrument, including administration time, number of items, translations to Scandinavian languages, and information on the psychometric properties investigated. Most (*n* = 78, 82%) of the 95 included studies were published in Europe, the USA or Canada, and publication years ranged from the 1990 s to 2024. The article types included prospective studies (*n* = 47, 49,4%), cross sectional studies or surveys (*n* = 38, 40%), systematic reviews (*n* = 8, 8.4%), and retrospective studies (*n* = 2, 2.1%).

Regarding area of concern, we found eighteen (72%) generic questionnaires back issues, two (8%) questionnaires related to general MSDs and, and one (4%) specifically developed for upper extremity disorders. The most commonly reported tools were the Fear-Avoidance Beliefs Questionnaire (FABQ) (*n* = 15 studies, 16%) and the Örebro Musculoskeletal Pain Questionnaire (OMPQ)—25 (*n* = 12 studies, 13%). All questionnaires, except for the Subjective Prognosis of Employability (SPE) [29] and the Erfassung von Funktionsfähigkeit im Beruf (SE-FFB) [30] were available in English. 10 tools (40%) were available in Scandinavian languages (Table 1, supplementary file) and these tools will be further presented in a future paper.

The tools comprised between 1 and 60 items and took between 1 and 20 min to complete.

A typical example of a questionnaire is the Örebro Musculoskeletal Pain Screening Questionnaire—short form (ÖMPQ-SF). It consists of 10 items and takes approximately 3–5 min to complete. This questionnaire addresses the body functions as well as the activity and participation dimensions of the ICF model in questions and statements like “ How would you rate the pain that you have had during the past week?” and “ I can do light work (or home duties) for an hour”. It has been well-validated and translated into multiple languages [54, 72–75].

### Measurement Properties

Most (n = 17, 68%) of the questionnaires included in this scoping review on patients with MSDs have been evaluated for multiple measurement properties, including validity, reliability, and predictive ability (Table 1). A total of 20 tools have been examined for validity for this specific patient group, with content validity and construct validity being the most frequently investigated aspects. Validation methods commonly included interviews, exploratory and confirmatory factor analyses, hypothesis testing, and correlation analyses. Three of the identified studies mentioned that the COSMIN checklist was followed in the validation process [72, 98, 101].

Predictive validity was assessed in 11 out of 25 identified tools, representing 44% (Table 1). The follow-up period for most studies ranged from 3 to 12 months. Some studies only included follow-up periods immediately after the intervention had concluded (2–10 weeks) [44, 70], while a few studies had follow-up periods of up to two years [47, 96] and one had a three-year follow-up period [56]. Additionally, 15 tools (60%) have been examined for reliability and seven (28%) for responsiveness. Three studies (12%) evaluated discriminant ability [54, 70, 121], and only two tools (8%), the Norwegian Function Assessment Scale (NFAS) and Work Ability Index (WAI), had established normative data [51, 103].

### Linkage to ICF Dimensions

Table 1 presents the different tools and their linkage to second level ICF codes. Looking at the ICF dimensions covered, 21 tools (84%) covered body functions, all tools (100%) covered activities and participation, 14 tools (56%) covered environmental factors, and 6 tools (24%) covered personal factors, which was related to age, sex, occupation and coping strategies. The Work Ability Score (WAS) [97] and the Subjective Prognosis Of Employability (SPE) [29] represented items from the activity and participation dimension only. Three tools (12%) covered all ICF dimensions: Readiness For Return To Work Scale (RRTW) [82], work rehabilitation questionnaire (WORQ) [113] and Quickscan Assesses Risk of Long-Term Sickness Absence [119].

**Table 1:** Linkage to ICF codes 1

Tool	Body functions and structure	Activity/Participation	Environment	Personal factors
Absenteeism Screening Questionnaire (ASQ)	b164: higher-level cognitive functions b280-b289: pain	d230: carrying out daily routine d350: conversation d410-d469: mobility d740: formal relationship d810-d839: education d840-d879: work and employment	e135: products and technology for employment e325: acquaintances, peers, colleagues, neighbours and community members e330: people in positions of authority e355: health professionals e430: individual attitudes of people in positions of authority e435: individual attitudes of people in subordinate positions	
Blue flags	b140: attention functions b164: higher-level cognitive functions	d175: solving problems d220: undertaking multiple tasks d240: handling stress and other psychological demands d845: acquiring, keeping and terminating a job d850: remunerative employment	e325: acquaintances, peers, colleagues, neighbours and community members e330: people in positions of authority e425: individual attitudes of acquaintances, peers, colleagues, neighbours and community members e430: individual attitudes of people in positions of authority	
Brief Screening Questionnaire	b280: pain	d230: carrying out daily routine d640: doing housework d840-d879: work and employment		
Fear-Avoidance Beliefs Questionnaire (FABQ)	b152: emotional functions b164: higher-level cognitive functions b280: pain	d410-d469: mobility d845: acquiring, keeping and terminating a job d850: remunerative employment		
Graded Reduced Work Ability Scale (GRWA)	b289: sensation of pain, other specified and unspecified	d230: carrying out daily routine d570: looking after one’s health d845: acquiring, keeping and terminating a job d850: remunerative employment		
Job Leeway Scale	b164: higher-level cognitive functions b289: sensation of pain, other specified and unspecified	d230: carrying out daily routine d240: handling stress and other psychological demands d570: looking after one’s health d410-469: mobility	e135: products and technology for employment e325: acquaintances, peers, colleagues, neighbours and community members	
Modified version of the Spinal Function Sort (M-SFS)		d410-469: mobility	e135: products and technology for employment e255: vibration	
Norwegian Function Assessment Scale (NFAS)	b126: temperament and personality functions b140: attention functions b144: memory functions b152: emotional functions b164: higher-level cognitive functions	d110: watching d115: listening d310-d369: communication d410-d469: mobility d470: using transportation d475: driving d540: dressing d630: preparing meals d640: doing housework d660: assisting others d920: recreation and leisure		Coping styles
Örebro Musculoskeletal Pain Questionnaire (OMPQ −25)/ Acute Low Back Pain Screening Questionnaire (ALBPSQ) Short versions: örebro Musculoskeletal Pain Screening Questionnaire-Short Form (OMPQ-SF-10) Örebro Musculoskeletal Screening Questionnaire (OMPQ)- 21 Örebro Musculoskeletal Screening Questionnaire (OMSQ -SV12)	b134: sleep functions b152: emotional functions b280: pain	d230: carrying out daily routine d410-469: mobility d640#: doing housework d850: remunerative employment d920¤: recreation and leisure		Sex, age
Pain Recovery Inventory of Concerns and Expectations (PRICE)	b152: emotional functions b147: psychomotor functions b160: thought functions b280: pain	d410-d469: mobility	e330: people in positions of authority	
Readiness for Return to Work Scale (RRTW)	b164: higher-level cognitive functions b455: exercise tolerance functions	d175: solving problems d230: carrying out daily routine d240: handling stress and other psychological demands d410-d469: mobility d570: looking after one’s health d845: acquiring, keeping and terminating a job d850: remunerative employment d920: recreation and leisure	e325: acquaintances, peers, colleagues, neighbours and community members e330: people in positions of authority	Coping strategies
Return-To-Work Self-Efficacy Scale Long version (RTWSE-19) Short version (RTWSE-10)	b289: sensation of pain, other specified and unspecified	d230: carrying out daily routine d410-469: mobility d570: looking after one’s health d740: using transportation d845: acquiring, keeping and terminating a job d850: remunerative employment	e325: acquaintances, peers, colleagues, neighbours and community members	
Risk Factor Questionnaire (RFQ)		d220: undertaking multiple tasks d230: carrying out daily routine d240: handling stress and other psychological demands d410-d469: mobility d475: driving d845: acquiring, keeping and terminating a job d850: remunerative employment	e135: products and technology for employment	
Scales For the Assessment of Functioning at Work (SE-FFB)	b130: energy and drive functions b140: attention functions b144: memory functions b152: emotional functions b164: higher-level cognitive functions b280: pain	d175: solving problems d230: carrying out daily routine d240: handling stress and other psychological demands d410-d469: mobility d475: driving d415: maintaning a body position d430: lifting and carrying objects d445: hand and arm use d470: using transportation d750: informal social relationship d845: acquiring, keeping and terminating a job d850: remunerative employment d910: community life		Coping strategies
Screening Instrument for Identification of a Demand for Medical-Vocational Oriented Rehabilitation (SIMBO)	b280: pain	d845: acquiring, keeping and terminating a job d850: remunerative employment	e325: acquaintances, peers, colleagues, neighbours and community members e355: health professionals	
Subjective Prognosis of Employability (SPE)		d298: General tasks and demands, other specified d845: acquiring, keeping and terminating a job d850: remunerative employment		
Work Ability Score (WAS)		d850: remunerative employment		
Work Ability Index (WAI)	b110- b189: mental functions b410-b469: heart and circulatory functions b510-b559: function of the digestive, metabolic and endocrine system b610-b679: genital and reproductive functions b710-b789: neuromusculoskeletal and movement-related functions b810-b869: function of the skin and related structures	d230: carrying out daily routine d240: handling stress and other psychological demands d455: moving around d845: acquiring, keeping and terminating a job d850: remunerative employment		
Work Functioning Impairment Scale (WFun)	b160: thought functions b164: higher-level cognitive functions	d163: thinking d230: carrying out daily routine d240: handling stress and other psychological demands d720: complex interpersonal interactions d845: acquiring, keeping and terminating a job d850: remunerative employment		
Work Limitations Questionnaire (WLQ-25) (WLQ-26)	b126: temperament and personality functions b140: attention functions b144: memory functions b152#: emotional functions, b160#: thought functions b289: sensation of pain, other specified and unspecified	d160#: focusing attention d166#: reading d220: undertaking multiple tasks d230: carrying out daily routine d240: handling stress and other psychological demands d350-69: Conversation and use of communication devices and techniques d410: Changing basic body position d415: Maintaining a body position d430: Lifting and carrying objects d435#: Moving objects with lower extremities d440#: fine hand use d445: hand and arm use d450: walking d455: moving around d660: Assisting household members and others d710#: Basic interpersonal interactions d810-d850: education and employment	e135: products and technology for employment e430*: individual attitudes of people in positions of authority	
Work Rehabilitation Questionnaire (WORQ)	b117: intellectual functions b126: Temperament and personality functions b130: Energy and drive functions b134: sleep functions b140: attention functions b144: memory functions b152: emotional functions, b160: thought functions b164: higher-level cognitive functions b210: seeing functions b230: hearing functions b235: vestibular functions b280: pain b455: exercise tolerance functions b730: muscle power functions b810: protective functions of the skin	d155: solving problems d160: focusing attention d166: reading d177: making decisions d210: undertaking a single task d330: speaking d230: carrying out daily routine d240: handling stress and other psychological demands d315: communicating with—receiving—nonverbal messages d350: conversation d360: using communication devices and techniques d410-d469: mobility d470-d475: using transportation and driving d540: dressing d570: looking after one’s health d720: Complex interpersonal interactions d810-d870: education and employment	e310: immediate family e330: people in positions of authority e355: health professionals e580: health services, systems and policies e590: labour and employment services, systems and policies	occupation
Work-Related Questionnaire for upper extremity disorders (WORQ-UP)	b710: mobility of joint functions b760: control of voluntary movement functions	d360: using communication devices and techniques d430: lifting and carrying objects d440: fine hand use d445: hand and arm use d455: moving around d470: using transportation d475: driving	e135: Products and technology for employment	
Work Role Functioning Questionnaire (WRFQ)	b130: energy and drive functions b140: attention functions b152: emotional functions	d220: undertaking multiple tasks d230: carrying out daily routine d240: handling stress and other psychological demands d350: conversation d360: using communication devices and techniques d410: changing basic body positions d430: lifting and carrying objects d440: fine hand use d445: hand and arm use d455: moving around d845: acquiring, keeping and terminating a job, d850: remunerative employment	e135: products and technology for employment e430: individual attitudes of people in positions of authority	
Quickscan Assesses Risk of Long-Term Sickness Absence	b126: temperament and personality functions b130: energy and drive functions b152: emotional functions b280: pain	d410-d469: mobility d570: looking after one’s health d845: acquiring, keeping and terminating a job d850: remunerative employment	e325: acquaintances, peers, colleagues, neighbours and community members e330: people in positions of authority e425: individual attitudes of acquaintances, peers, colleagues, neighbours and community members e585: education and training services, systems and policies	occupation
3 questions for estimating the risk of no RTW	b280: pain	d230: carrying out daily routine d850: remunerative employment		

^1^ Not all all numbers correspond to the number of items in the tool, due to lack of linkage to ICF-codes, or one item representing more than one code

^*^ Short version, #Long version ¤ Screening version

## Discussion

This scoping review aimed to achieve a comprehensive overview of available tools for assessing work ability in workers with musculoskeletal disorders. In total, 25 different questionnaires were identified, and 10 were available in either Norwegian, Swedish or Danish. To our knowledge, this is the first review that focuses on tools designed to assess work ability suitable for primary healthcare and in the early phase of sickness absence for individuals with MSDs, as recommended by health authorities [7]. This unique focus is vital for providing primary care clinicians with reliable and practical tools to support early interventions that can potentially expedite return to work and improve work participation outcomes for patients with MSDs.

A diverse range of tools were included in this review, varying significantly in their content and utility, with the number of items ranging from 1 to 60. This variety reflects the different definitions and conceptualizations of work ability, and the different objectives these tools aim to achieve. Some tools consisted of a single question, while others were comprehensive forms addressing numerous factors related to work ability. One approach to utilizing these questionnaires could be to begin with a simple question, such as the Work Ability Scale (WAS) (1st question of the Work Ability Index, WAI):"What is your current work ability compared to your lifetime best?". This single question has shown efficacy in assessing the current level and progression of work ability [122]. However, it does not provide guidance on interventions to improve work ability but can serve as a screening tool or a starting point for further discussion about the work situation. To achieve a comprehensive understanding of work ability, it is essential to consider insights into patients’ beliefs, motivation, and perceptions of health, and the working environment [8, 9]. In situations where following up patients with prolonged sick leave or in complex cases, it may be appropriate to utilize a more detailed questionnaire, such as the Absenteeism Screening Questionnaire (ASQ). Our findings demonstrate that both generic and disease specific questionnaires are available for assessing work ability in individuals with MSDs seeking help. Generic questionnaires have broad application across different types and severity of diseases, making them accessible for various professionals, including those who may not specialise in MSDs. Their wide applicability across various populations makes them advantageous for large-scale screening and research initiatives [123]. A key argument for utilizing generic questionnaires is their ability to look beyond specific health conditions and diagnoses, focusing more on the individual and their resources, rather than letting the condition define the worker. However, generic instruments may fall outside the perceived responsibility and competence area of healthcare professionals, providing less precise guidance on assisting employees in returning to or remaining in their jobs.

Disease specific questionnaires provide detailed, condition-specific insights allowing for a more nuanced understanding of how the disorder impacts work ability. They may be more useful in evaluating clinical management and limitations associated with specific disease conditions. For instance, they may highlight specific functional impairments, pain patterns, or psychological barriers associated with particular MSDs, which can be crucial for developing effective rehabilitation strategies [123].

## Relation to ICF Dimensions

The studies included in this scoping review show that the most frequently covered ICF dimensions were activities and participation. This aligns with findings from the study by Sternberg et al. [20], who found that workplace characteristics, the worker’s social environment, and broader societal and personal factors receive relatively less attention. The causes of reduced work ability in patients with MSDs are complex and multifactorial [124, 125]. In accordance with the biopsychosocial model, integrating overall functions, psychosocial factors, and social consequences should be considered early in the return-to-work process to identify factors associated with reduced work ability [21]. Our findings identified only three questionnaires (Readiness For Return To Work Scale (RRTW) [82], work rehabilitation questionnaire (WORQ) [113] and Quickscan Assesses Risk of Long-Term Sickness Absence [119]) that comprehensively covered all dimensions of the ICF. This gap highlights the importance for clinicians and researchers to be aware of which tools they choose when they aim to assess both individual capacities and the broader work context.

## Relevance of Assessments for Return to Work

In our study, we included only self-reported tools. Previous studies [122, 126] have demonstrated that an individual’s self-report on work ability is a significant predictor of future sickness absence. Individuals often possess a more holistic understanding of what affects their work ability, particularly concerning non-medical factors [122]. However, self-reports may have the potential for errors such as social desirability bias, misinterpretation of questions, and respondents might lack the ability to accurately assess their own behaviors or feelings. Further, self-reports may be at risk of respondent bias, as some respondents may tend to agree with statements regardless of their content. In the case of comprehensive questionnaires with a long list of questions, respondents might also experience fatigue, leading to rushed or incomplete answers. Performance based tests are widely used to evaluate work ability and are often recommended alongside self-reported questionnaires, as they measure different yet related aspects [13]. Combining self-report and performance-based tests improves prognostic values for sustainable return to work [105] and should be considered, as it may increase the overall face validity of the evaluation [127].

## Clinimetric Quality

Robust psychometric properties are essential for providing accurate and reliable assessments of work ability. We aimed to provide an overview of the properties that were evaluated for each tool in the context of workers with MSD. The tools we identified were assessed for one or more psychometric properties, but the details and strength of each property and each tool were beyond the scope of this review.

Tools like the Örebro Musculoskeletal Pain Questionnaire (both long and short versions), and the Fear-Avoidance Beliefs Questionnaire (FABQ) are questionnaires developed for patients with MSDs and have been examined for various measurement properties in numerous studies. Additionally, they have also been translated, culturally adapted, and validated in several languages, contributing to the robustness and wide applicability [128–138]. Other tools, like the Work Ability Index (WAI) and the Work Rehabilitation Questionnaire (WORQ), are generic questionnaires that have been examined for measurement properties across various working groups and patient groups, but to a lesser extent have been examined for individuals with MSD in the early phase of sickness absence [140–144]. Our results identified one recently developed tool, the Job Leeway Scale [49], which the measurement properties (validity and reliability) were examined in a single study. Nevertheless, it is likely to undergo further validation in the future.

Despite being developed many years ago, tools such as the Modified version of the Spinal Function Sort (M-SFS), Subjective Prognosis of Employability (SPE), and Risk Factor Questionnaire (RFQ), may have limited applicability for patients with MSDs due to the lack of studies examining psychometric properties in this specific population. The discriminative ability and normative data can also be useful for clinicians to distinguish and identify workers at risk of sickness absence and to understand where an individual stands relative to a broader population. Unfortunately, these properties were only available for a few tools. Studies on discriminant ability was found for the tools The back specific version of the OMPQ: Acute Low Back Pain Screening Questionnaire (ALBPSQ) [70], Norwegian Function Assessment Scale (NFAS) [54], Örebro Musculoskeletal Pain Questionnaire (OMPQ-SF – 10) [54] and 3 questions for estimating the risk of no RTW [121]. Normative data was found for the Norwegian Function Assessment Scale (NFAS) [51] and Work Ability Index (WAI) [103]. Accordingly, these properties should be subject to future studies.

## Strength and Limitations

The strengths of this study lie in the employment of rigorous and transparent methods throughout the entire research process. The study was conducted according to a published protocol and adhered to the PRISMA extension for Scoping Reviews (PRISMA-ScR) as a guiding checklist (supplementary material). To ensure comprehensive management of citations and articles, we utilized a bibliographic manager (EndNote) and systematic review software (DistillerSR).

Although other reviews have already investigated tools to assess work ability [15, 20, 21], the strength and justification of this review is the specific focus on the critical phase of early sickness absence or workers at risk for sickness absence. Further, by selecting time-efficient tools, we provide clinicians with feasible instruments in busy clinical practice in primary health care. In addition, we expanded the inclusion criteria to include studies regardless of whether they had references to the ICF, and we did not restrict data inclusion by language or date. Focusing exclusively on self-reported questionnaires limits our study to assessment at an individual level. It only partially addresses the factors of work organization, conditions, and legislation that are shaped by workplace and societal stakeholders, all of which are essential components of work ability. In addition, we narrowed the target tools to those that were either generic or developed for non-specific MSDs, omitting studies using tools developed for specific MSDs or conditions. Hence, we do not provide evidence regarding the applicability to workers with other health-related conditions. A final limitation of our scoping review is the lack of examination of digital versions of questionnaires or screening programs, which could provide valuable information for clinical practitioners and facilitate easier implementation of these tools.

## Concluding Remarks

The evidence base for work ability assessment tools is extensive, with significant variation in tools, linkage to ICF, and psychometric properties. This review identified few questionnaires covering all ICF dimensions. Less comprehensive questionnaires may suffice for initial screening, while full assessments are necessary for complex cases or prolonged absence to support sustainable return to work. Future research should explore the efficacy of digital tools and algorithms for screening based on self-reported questionnaires and further examine measurement properties like responsiveness and normative data.

The findings from this study are not only relevant for healthcare professionals assessing and guiding work ability among workers with MSD, but also for policymakers and stakeholders involved in developing work ability and return to work policies.

## Supplementary Information

Below is the link to the electronic supplementary material.

Supplementary file1 (PDF 289 KB)

Supplementary file2 (PDF 284 KB)

Supplementary file3 (PDF 211 KB)

Supplementary file4 (DOCX 34 KB)

## Data Availability

No datasets were generated or analysed during the current study.
