# Supplementary material for: Assessment of Work Ability in People with Musculoskeletal Disorders in Primary Health Care: A Scoping Review of Self-Reported Tools
Source: J Occup Rehabil. 2025 Nov 13;36(4):1001–19. doi: 10.1007/s10926-025-10323-1 (PMC13759573; doi:10.1007/s10926-025-10323-1)
Supplement: Supplementary file 1 [file 10926_2025_10323_MOESM1_ESM.pdf]

Search history – Final search update

Search performed 12. March 2025

Contents

Search strategy 1

EMBASE 2

Medline 3

AMED 4

CINAHL 5

Duplicate detection and removal 6

Search strategy

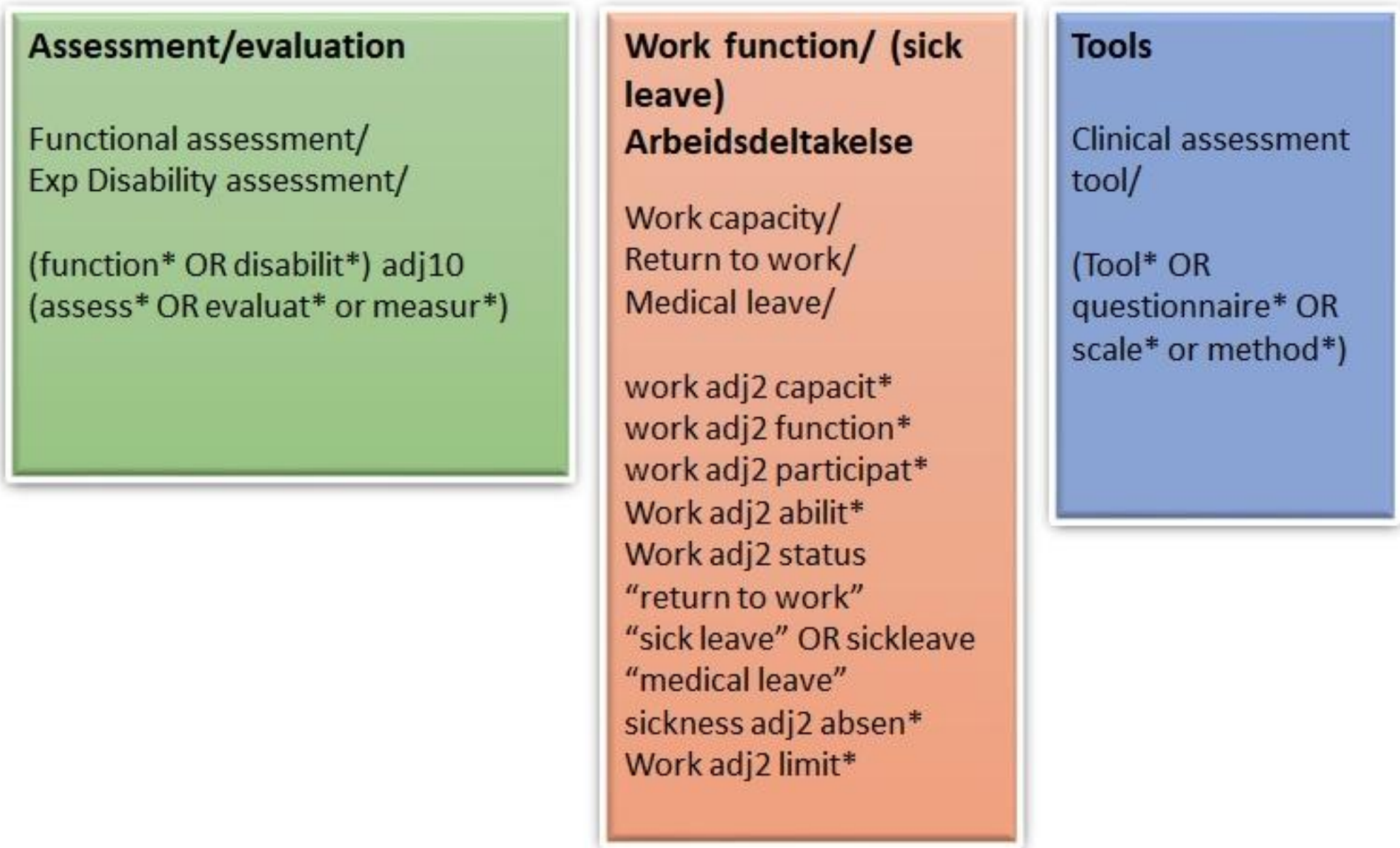

EMBASE

https://ovidsp.ovid.com/ovidweb.cgi?T=JS&NEWS=N&PAGE=main&SHAREDSEARCHID=sayvUbFe2gY3GUNe4yl4ynRKpLdTI5D6wuZaEdcuLGSAK61iCQTIilAP8e3p1ZCE

Embase <1974 to 2025 March 11>

1	functional assessment/	74827
2	exp disability assessment/	62157
3	((function* or disabilit*) adj10 (assess* or evaluat* or measur*)).ab,kf,ti.	873658
4	1 or 2 or 3	953466
5	return to work/	11217
6	work capacity/	14464
7	medical leave/	9506
8	(work adj2 capacit*).ab,kf,ti.	6774
9	(work adj2 function*).ab,kf,ti.	9144
10	(work adj2 participat*).ab,kf,ti.	2277
11	(work adj2 abilit*).ab,kf,ti.	8302
12	(work adj2 status).ab,kf,ti.	5496
13	"return to work".ab,kf,ti.	16598
14	("sick leave" or sickleave).ab,kf,ti.	9011
15	"medical leave".ab,kf,ti.	474
16	(sickness adj2 absen*).ab,kf,ti.	4342
17	(work adj2 limit*).ab,kf,ti.	5367
18	5 or 6 or 7 or 8 or 9 or 10 or 11 or 12 or 13 or 14 or 15 or 16 or 17	73607
19	"tool*".ab,kf,ti.	1497938
20	"questionnaire*".ab,kf,ti.	1075028
21	clinical assessment tool/	29411
22	"scale*".ab,kf,ti.	1647099
23	"method*".ab,kf,ti.	13677044
24	19 or 20 or 21 or 22 or 23	15188416
25	4 and 18 and 24	6589
26	limit 25 to yr="2023 -Current"	837

Medline

https://ovidsp.ovid.com/ovidweb.cgi?T=JS&NEWS=N&PAGE=main&SHAREDSEARCHID=3tV0ZePu9qiatXzKzznJSdIulQeBtfUIBs4qo4OJnu8Y7nnAW8cijgEF8USlKXBY

Ovid MEDLINE(R) ALL <1946 to March 11, 2025>

1	exp disability evaluation/	58187
2	((function* or disabilit*) adj10 (assess* or evaluat* or measur*)).ab,kf,ti.	607932
3	1 or 2	646629
4	return to work/	4083
5	sick leave/	7119
6	(work adj2 capacit*).ab,kf,ti.	6187
7	(work adj2 function*).ab,kf,ti.	8972
8	(work adj2 participat*).ab,kf,ti.	1750
9	(work adj2 abilit*).ab,kf,ti.	6020
10	(work adj2 status).ab,kf,ti.	4020
11	"return to work".ab,kf,ti.	12712
12	("sick leave" or sickleave).ab,kf,ti.	6951
13	"medical leave".ab,kf,ti.	380
14	(sickness adj2 absen*).ab,kf,ti.	3751
15	(work adj2 limit*).ab,kf,ti.	4184
16	4 or 5 or 6 or 7 or 8 or 9 or 10 or 11 or 12 or 13 or 14 or 15	51546
17	"tool*".ab,kf,ti.	1143348
18	"questionnaire*".ab,kf,ti.	757438
19	"scale*".ab,kf,ti.	1272910
20	"method*".ab,kf,ti.	9260693
21	17 or 18 or 19 or 20	10605007
22	3 and 16 and 21	5781
23	limit 22 to yr="2023 -Current"	621

AMED

https://ovidsp.ovid.com/ovidweb.cgi?T=JS&NEWS=N&PAGE=main&SHAREDSEARCHID=3QVj9dGwcf9uUagut0XPWWSsuumMnRLAHcg46c5mkouYGqUtuynoVpiphP84Oe3RR

AMED (Allied and Complementary Medicine) <1985 to February 2025>

1	exp disability evaluation/	8552
2	((function* or disabilit*) adj10 (assess* or evaluat* or measur*)).ab,ti.	20132
3	1 or 2	25306
4	sick leave/	195
5	(work adj2 capacit*).ab,ti.	305
6	(work adj2 function*).ab,ti.	285
7	(work adj2 participat*).ab,ti.	208
8	(work adj2 abilit*).ab,ti.	321
9	(work adj2 status).ab,ti.	274
10	"return to work".ab,ti.	1206
11	("sick leave" or sickleave).ab,ti.	398
12	"medical leave".ab,ti.	6
13	(sickness adj2 absen*).ab,ti.	132
14	(work adj2 limit*).ab,ti.	171
15	4 or 5 or 6 or 7 or 8 or 9 or 10 or 11 or 12 or 13 or 14	2723
16	"tool*".ab,ti.	9703
17	clinical assessment scales/	4922
18	"questionnaire*".ab,ti.	16677
19	"scale*".ab,ti.	21398
20	"method*".ab,ti.	82566
21	16 or 17 or 18 or 19 or 20	103564
22	3 and 15 and 21	526
23	limit 22 to yr="2023 -Current"	0

CINAHL

Expanders – Apply equivalent subjects
Saerch modes Proximity

S1	(MH "Functional Assessment")	21,461
S2	(MH "Disability Evaluation+")	18,561
S3	TI ((function* OR disabilit*) N9 (assess* OR evaluat* or measur*)) OR AB ((function* OR disabilit*) N9 (assess* OR evaluat* or measur*)) OR SU ((function* OR disabilit*) N9 (assess* OR evaluat* or measur*))	181,135
S4	S1 OR S2 OR S3	182,461
S5	(MH "Job Re-Entry")	7,953
S6	(MH "Sick Leave")	5,596
S7	TI work N1 capacit* OR AB work N1 capacit* OR SU work N1 capacit*	2,849
S8	TI work N1 function* OR AB work N1 function* OR SU work N1 function*	1,232
S9	TI work N1 participat* OR AB work N1 participat* OR SU work N1 participat*	1,137
S10	TI Work N1 abilit* OR AB Work N1 abilit* OR SU Work N1 abilit*	2,489
S11	TI Work N1 status OR AB Work N1 status OR SU Work N1 status	1,883
S12	TI "return to work" OR AB "return to work" OR SU "return to work"	6,190
S13	TI "medical leave" OR AB "medical leave" OR SU "medical leave"	868
S14	TI sickness N1 absen* OR AB sickness N1 absen* OR SU sickness N1 absen*	1,956
S15	(TI work N1 limit* OR AB work N1 limit* OR SU work N1 limit*) OR (TI ("sick leave" OR sickleave) OR AB ("sick leave" OR sickleave) OR SU ("sick leave" OR sickleave))	8,244
S16	S5 OR S6 OR S7 OR S8 OR S9 OR S10 OR S11 OR S12 OR S13 OR S14 OR S15	25,884
S17	(MH "Clinical Assessment Tools+")	290,103
S18	TI (tool* OR questionnaire* OR scale* OR method*) OR AB (tool* OR questionnaire* OR scale* OR method*) OR SU (tool* OR questionnaire* OR scale* OR method*)	3,013,783
S19	S17 OR S18	3,043,279
S20	S4 AND S16 AND S19	3,319
S21	S4 AND S16 AND S19	301

Duplicate detection and removal

1759 references were imported from the period 2023-current. After duplicates were removed 1158 references remained. These references were then compared to the 9591 references from the original search to look for overlap. A total of 931 new referencse were finally added to the DistillerSR project.
