# Supplementary material for: Assessment of Work Ability in People with Musculoskeletal Disorders in Primary Health Care: A Scoping Review of Self-Reported Tools
Source: J Occup Rehabil. 2025 Nov 13;36(4):1001–19. doi: 10.1007/s10926-025-10323-1 (PMC13759573; doi:10.1007/s10926-025-10323-1)
Supplement: Supplementary file 2 [file 10926_2025_10323_MOESM2_ESM.pdf]

Search history – Final search

Search performed 15th of June 2023

Contents

Search strategy 1

EMBASE 2

Medline.. 3

AMED... 4

CINAHL... 5

PEDro (Physiotherpay Evidence Database)... 6

Search strategy

Assessment/evaluation

Functional assessment/
Exp Disability assessment/

(function* OR disabilit*) adj10 (assess* OR evaluat* or measur*)

Work function/ (sick leave) Arbeidsdeltakelse

Work capacity/
Return to work/
Medical leave/

work adj2 capacit*
work adj2 function*
work adj2 participat*
Work adj2 abilit*
Work adj2 status
“return to work”
“sick leave” OR sickleave
“medical leave”
sickness adj2 absen*
Work adj2 limit*

Tools

Clinical assessment tool/

(Tool* OR questionnaire* OR scale* or method*)

EMBASE

Embase <1974 to 2023 Week 23>

1	functional assessment/	71080
2	exp disability assessment/	45520
3	((function* or disabilit*) adj10 (assess* or evaluat* or measur*)).ab,kf,ti.	797571
4	1 or 2 or 3	865209
5	work capacity/	13665
6	return to work/	9672
7	medical leave/	8669
8	(work adj2 capacit*).ab,kf,ti.	6463
9	(work adj2 function*).ab,kf,ti.	7899
10	(work adj2 participat*).ab,kf,ti.	1998
11	(work adj2 abilit*).ab,kf,ti.	7413
12	(work adj2 status).ab,kf,ti.	5007
13	"return to work".ab,kf,ti.	15279
14	("sick leave" or sickleave).ab,kf,ti.	8377
15	"medical leave".ab,kf,ti.	412
16	(sickness adj2 absen*).ab,kf,ti.	4092
17	(work adj2 limit*).ab,kf,ti.	4796
18	5 or 6 or 7 or 8 or 9 or 10 or 11 or 12 or 13 or 14 or 15 or 16 or 17	67226
19	clinical assessment tool/	27570
20	"tool*".ab,kf,ti.	1316798
21	"questionnaire*".ab,kf,ti.	967803
22	"scale*".ab,kf,ti.	1459765
23	"method*".ab,kf,ti.	12280073
24	19 or 20 or 21 or 22 or 23	13669232
25	4 and 18 and 24	5875

Medline

Ovid MEDLINE(R) and Epub Ahead of Print, In-Process, In-Data-Review & Other Non-Indexed Citations and Daily <1946 to June 14, 2023>

1	exp disability evaluation/	56347
2	((function* or disabilit*) adj10 (assess* or evaluat* or measur*)).ab,kf,ti.	544120
3	1 or 2	581947
4	return to work/	3631
5	sick leave/	6750
6	(work adj2 capacit*).ab,kf,ti.	5868
7	(work adj2 function*).ab,kf,ti.	7637
8	(work adj2 participat*).ab,kf,ti.	1499
9	(Work adj2 abilit*).ab,kf,ti.	5283
10	(Work adj2 status).ab,kf,ti.	3603
11	"return to work".ab,kf,ti.	11485
12	("sick leave" or sickleave).ab,kf,ti.	6365
13	"medical leave".ab,kf,ti.	336
14	(sickness adj2 absen*).ab,kf,ti.	3446
15	(work adj2 limit*).ab,kf,ti.	3614
16	4 or 5 or 6 or 7 or 8 or 9 or 10 or 11 or 12 or 13 or 14 or 15	46356
17	"tool*".ab,kf,ti.	973080
18	"questionnaire*".ab,kf,ti.	664762
19	"scale*".ab,kf,ti.	1097659
20	"method*".ab,kf,ti.	8148545
21	17 or 18 or 19 or 20	9347882
22	3 and 16 and 21	5327

AMED

AMED (Allied and Complementary Medicine) <1985 to May 2023>

1	exp disability evaluation/	8530
2	((function* or disabilit*) adj10 (assess* or evaluat* or measur*)).ti,ab.	19607
3	1 or 2	24771
4	sick leave/	195
5	(work adj2 capacit*).ti,ab.	303
6	(work adj2 function*).ti,ab.	279
7	(work adj2 participat*).ti,ab.	202
8	(work adj2 abilit*).ti,ab.	318
9	(work adj2 status).ti,ab.	273
10	"return to work".ti,ab.	1182
11	("sick leave" or "sickleave").ti,ab.	394
12	"medical leave".ti,ab.	6
13	(sickness adj2 absen*).ti,ab.	130
14	(work adj2 limit*).ti,ab.	166
15	4 or 5 or 6 or 7 or 8 or 9 or 10 or 11 or 12 or 13 or 14	2678
16	clinical assessment scales/	4918
17	"tool*".ti,ab.	9316
18	"questionnaire*".ti,ab.	16298
19	"scale*".ti,ab.	20698
20	"method*".ti,ab.	79669
21	16 or 17 or 18 or 19 or 20	100316
22	3 and 15 and 21	523

CINAHL

#	Query	Results
S1	(MH "Functional Assessment")	19,509
S2	(MH "Disability Evaluation+")	18,271
S3	TI ((function* OR disabilit*) N9 (assess* OR evaluat* or measur*)) OR AB ((function* OR disabilit*) N9 (assess* OR evaluat* or measur*)) OR SU ((function* OR disabilit*) N9 (assess* OR evaluat* or measur*))	171,572
S4	S1 OR S2 OR S3	172,766
S5	(MH "Job Re-Entry")	7,412
S6	(MH "Sick Leave")	5,316
S7	TI work N1 capacit* OR AB work N1 capacit* OR SU work N1 capacit*	2,651
S8	TI work N1 function* OR AB work N1 function* OR SU work N1 function*	1,157
S9	TI work N1 participat* OR AB work N1 participat* OR SU work N1 participat*	1,034
S10	TI Work N1 abilit* OR AB Work N1 abilit* OR SU Work N1 abilit*	2,308
S11	TI Work N1 status OR AB Work N1 status OR SU Work N1 status	1,759
S12	TI "return to work" OR AB "return to work" OR SU "return to work"	5,882
S13	TI ("sick leave" OR sickleave) OR AB ("sick leave" OR sickleave) OR SU ("sick leave" OR sickleave)	6,580
S14	TI "medical leave" OR AB "medical leave" OR SU "medical leave"	836
S15	TI sickness N1 absen* OR AB sickness N1 absen* OR SU sickness N1 absen*	1,938
S16	TI work N1 limit* OR AB work N1 limit* OR SU work N1 limit*	1,308
S17	S5 OR S6 OR S7 OR S8 OR S9 OR S10 OR S11 OR S12 OR S13 OR S14 OR S15 OR S16	24,476
S18	(MH "Clinical Assessment Tools+")	284,705
S19	TI (tool* OR questionnaire* OR scale* OR method*) OR AB (tool* OR questionnaire* OR scale* OR method*) OR SU (tool* OR questionnaire* OR scale* OR method*)	2,854,862
S20	S4 AND S17 AND S19	3,057

PEDro (Physiotherpay Evidence Database)

A systematic search, like the one we have performed in the previous databases, is not supported in PEDro. Some searches combining words from the search strategy was carried out, but no relevant hits were found. This is not surprising, considering PEDro is a database of randomised controlled trials, systematic reviews and clinical practice guidelines.

Duplicate detection and removal

14782 references were imported to EndNote for the removal of duplicates. 9591 references where then imported in DistillerSR for screening.
