# Supplementary material for: Assessment of Work Ability in People with Musculoskeletal Disorders in Primary Health Care: A Scoping Review of Self-Reported Tools
Source: J Occup Rehabil. 2025 Nov 13;36(4):1001–19. doi: 10.1007/s10926-025-10323-1 (PMC13759573; doi:10.1007/s10926-025-10323-1)
Supplement: Supplementary file 4 [file 10926_2025_10323_MOESM4_ESM.docx]

Table 1. Overview of tools and their characteristics

| **Tool** | **Time to administer (min)** | **Number of items, (n)** | **Measurement properties examined** | **Area of concern** |
| --- | --- | --- | --- | --- |
| Absenteeism Screening Questionnaire (ASQ) | 5-10 min | 22 | Validity [1]  Predictive ability [1] | Back |
| Blue flags* | 5-10 min | 14 | Validity [2] | Generic |
| Brief Screening Questionnaire | 1-2 minutes | 3 | Predictive ability [3] | Back |
| Fear-Avoidance Beliefs Questionnaire (FABQ)* | 5-10 min | 16 | Reliability [4-8]  Validity [4-12]  Predictive ability [7, 10, 13-18]  Responsiveness [5] | Back |
| Graded Reduced Work Ability scale (GRWA)* | 3-5 min | 6 | Reliability [18, 19] | Generic |
| Job Leeway scale | 5-10 min | 18 | Reliability [20]  Validity [20] | Generic |
| Modified version of the spinal function sort  (M-SFS) | 5-10 min | 20 | Validity [21] | Back |
| Norwegian Function Assessment Scale (NFAS)* | 10-15 min | 39 | Normative data [22]  Reliability [22]  Validity [23, 24]  Discriminant ability [25] | General MSD |
| Örebro Musculoskeletal Pain Questionnaire  (OMPQ) – 25* / Acute Low Back Pain Screening Questionnaire  (ALBPSQ)*  Örebro Musculoskeletal Pain  Screening Questionnaire-short form  (OMPQ-SF) – 10*  Örebro Musculoskeletal Screening Questionnaire  (OMPQ)- 21  Örebro Musculoskeletal Screening Questionnaire (OMSQ) -short version 12 | 5-10 min  3-5 min  5-10 min  3-5 min | 25 / 20  10  21  12 | Validity [26-29]  Predictive ability [26, 27, 29-37] / Validity [38]  Reliability [38, 39] Predictive ability [33, 38-42]  Discriminant ability [41]  Reliability [43]  Validity [43, 44]  Predictive ability [44-46]  Discriminant ability [25]  Reliability [47]  Validity [47]  Predictive ability [48]  Validity [49]  Predictive ability [49] | General MSD/ Back  General MSD  General MSD  General MSD |
| Pain Recovery Inventory of Concerns and Expectations  (PRICE) | 10-15 min | 46 | Reliability [50]  Validity [50]  Predictive ability [50] | Generic |
| Readiness for return to work scale (RRTW)* | 5-10 min | 22 | Reliability [51]  Validity [51-54] | Generic |
| Return-to-work self-efficacy scale (RTWSE-19) *  Return-To-Work Self-Efficacy scale (RTWSE-10) | 5-10 min  3-5 min | 19  10 | Reliability [55, 56]  Validity [55-57]  Predictive ability [18, 55, 58]  Responsiveness [56]  Validity [59, 60]  Predictive ability [61] | Generic  Generic |
| Risk factor questionnaire  (RFQ) | 5-10 min | 25 | Reliability [62] | Generic |
| Erfassung von Funktionsfähigkeit im Beruf (Scales for the Assessment of Functioning at Work)  (SE-FFB) | 5-10 min | 48 | Validity [63, 64]  Responsiveness [63] | Generic |
| Screening-Instrument zur Einschätzung des Bedarfs an Medizinisch-Beruflich Orientierten Maßnahmen in der medizinischen Rehabilitation (Screening Instrument for Identification of a Demand for Medical-Vocational Oriented Rehabilitation)  (SIMBO) | 3-5 min | 7 | Reliability [65]  Validity [65, 66]  Predictive ability [66-68] | Generic |
| Subjective prognosis of employability (SPE) | 1-2 min | 3 | Predictive ability [69, 70] | Generic |
| Work ability score  (WAS) | 1 min | 1 | Reliability [71]  Validity [71-73]  Predictive ability [74]  Responsiveness [71-73] | Generic |
| Work Ability Index  (WAI)* | 10-15 min | 7  (11 questions) | Reliability [75]  Validity [75, 76]  Normative data [77]  Predictive ability [18, 78, 79] | Generic |
| Work Functioning Impairment Scale (WFun) | 1-3 min | 7 | Validity [80]  Responsiveness [80] | Generic |
| Work Limitations Questionnaire  (WLQ-25)  Work Limitations Questionnaire  (WLQ-16) | 5-15 min  5-10 min | 25  16 | Reliability [81]  Validity [63, 81-84]  Responsiveness [63, 81, 84]  Reliability [85]  Validity [85, 86]  Responsiveness [86] | Generic  Generic |
| Work Rehabilitation Questionnaire (WORQ)* | 10-15 min | 40 | Reliability [87, 88]  Validity [87-89] | Generic |
| WOrk-Related Questionnaire for UPper extremity disorders  (WORQ-UP) | 5-10 min | 17 | Validity [90, 91] | Upper extremities |
| Work Role Functioning Questionnaire (WRFQ) * | 10-15 minutes | 27 | Reliability [81]  Validity [63, 81, 92]  Responsiveness [63, 81] | Generic |
| Quickscan Assesses Risk of Long-Term Sickness Absence | 15-20 minutes | 60 | Validity [93, 94] | Generic |
| 3 questions for estimating the risk of no RTW | 1 | 3 | Predictive ability [95]  Discriminant ability [95] | Generic |

*Available in a Scandinavian language

1.	Truchon, M., et al., *Absenteeism Screening Questionnaire (ASQ): A New Tool for Predicting Long-term Absenteeism Among Workers with Low Back Pain.* Journal of Occupational Rehabilitation, 2012. **22**(1): p. 27–50.

2.	Post Sennehed, C., et al., *“Blue flags”, development of a short clinical questionnaire on work-related psychosocial risk factors-a validation study in primary care.* BMC musculoskeletal disorders, 2017. **18**: p. 1–11.

3.	Fulton-Kehoe, D., et al., *Development of a brief questionnaire to predict long-term disability.* Journal of Occupational & Environmental Medicine, 2008. **50**(9): p. 1042–1052.

4.	Inrig, T., et al., *Validity and reliability of the Fear-Avoidance Beliefs Questionnaire (FABQ) in workers with upper extremity injuries.* Journal of occupational rehabilitation, 2012. **22**(1): p. 59–70.

5.	Grotle, M., Brox, JI. and Vøllestad, NK., *Reliability, validity and responsiveness of the fear-avoidance beliefs questionnaire: methodological aspects of the Norwegian version.* Journal of rehabilitation medicine, 2006. **38**(6): p. 346–353.

6.	Cleland, JA., Fritz, JM. and Childs, JD., *Psychometric properties of the Fear-Avoidance Beliefs Questionnaire and Tampa Scale of Kinesiophobia in patients with neck pain.* American journal of physical medicine & rehabilitation, 2008. **87**(2): p. 109–117

7.	Mintken, P.E., et al., *Psychometric properties of the Fear-Avoidance Beliefs Questionnaire and Tampa Scale of Kinesiophobia in patients with shoulder pain.* Archives of physical medicine and rehabilitation, 2010. **91**(7): p. 1128–1136.

8.	George, SZ., C. Valencia, and Beneciuk, JM., *A psychometric investigation of fear-avoidance model measures in patients with chronic low back pain.* Journal of orthopaedic & sports physical therapy, 2010. **40**(4): p. 197–205.

9.	Franchignoni, F., et al., *A further Rasch analysis of the Fear-Avoidance Beliefs Questionnaire in adults with chronic low back pain suggests the revision of its rating scale.* European journal of physical and rehabilitation medicine, 2021. **57**(1): p. 110–119.

10.	Fritz, JM., George S,Z, and Delitto, A., *The role of fear-avoidance beliefs in acute low back pain: relationships with current and future disability and work status.* Pain, 2001. **94**(1): p. 7–15.

11.	Lee, K.C., Chiu, TT and Lam, TH.,*The role of fear-avoidance beliefs in patients with neck pain: relationships with current and future disability and work capacity.* Clinical Rehabilitation, 2007. **21**(9): p. 812–21.

12.	Svensen, AR., Ringvold, MLT and Bergland, A., *Acute low back pain–a cross sectional study: Fear-avoidance beliefs and associated characteristics.* 2011

13.	Fritz, JM. and George, SZ., *Identifying psychosocial variables in patients with acute work-related low back pain: the importance of fear-avoidance beliefs.* Physical Therapy, 2002. **82**(10): p. 973–83.

14.	Holden, J., Davidson, M. and Tam, J., *Can the Fear-Avoidance Beliefs Questionnaire predict work status in people with work-related musculoskeletal disorders?* Journal of back and musculoskeletal rehabilitation, 2010. **23**(4): p. 201–8.

15.	Trinderup, J.S., et al., *Fear avoidance beliefs as a predictor for long-term sick leave, disability and pain in patients with chronic low back pain.* BMC Musculoskeletal Disorders, 2018. Dec 3;**19**(1):431.

16.	Steenstra, I., et al., *Systematic Review of Prognostic Factors for Return to Work in Workers with Sub Acute and Chronic Low Back Pain.* Journal of Occupational Rehabilitation, 2017. **27**(3): p. 369–381.

17.	Wertli, M.M., et al., *The role of fear avoidance beliefs as a prognostic factor for outcome in patients with nonspecific low back pain: a systematic review.* The spine journal, 2014. **14**(5): p. 816–836. e4.

18.	Villotti, P., et al., *Systematic review and tools appraisal of prognostic factors of return to work in workers on sick leave due to musculoskeletal and common mental disorders.* PLoS One, 2024. **19**(7): p. e0307284.

19.	Haldorsen, EM., Indahl, A. and Ursin, H., *Patients With Low Back Pain Not Returning to Work.* Spine (03622436), 1998. **23**: p. 1202–1207.

20.	Shaw, W.S., et al., *The Job Leeway scale: Initial evaluation of a self-report measure of health-related flexibility and latitude at work.* Journal of Occupational Rehabilitation, 2023. **33**(3): p. 581–591.

21.	Janssen, S., et al., *Development of a modified version of the spinal function sort (M-SFS): a mixed method approach.* Journal of occupational rehabilitation, 2016. **26**(3): p. 253–263.

22.	Østeras, N., et al., *Functional ability in a population: Normative survey data and reliability for the ICF based Norwegian Function Assessment Scale.* BMC Public Health, 2007. **7**: p. 278.

23.	Brage, S., et al., Norsk Funksjonsskjema-et nytt instrument ved sykmelding og uforhetsvurdering *[Norwegian Functional Scale--a new instrument in sickness certification and disability assessments]*, Tidsskr Nor Lægeforen 2004. **124**(19): 2472–2474.

24.	Østerås, N., et al., *A randomised comparison of a four- and a five-point scale version of the Norwegian Function Assessment Scale.* Health & Quality of Life Outcomes, 2008. **6**: p. 14–14.

25.	Ask, T., et al., *Self-Reported and Tested Function in Health Care Workers with Musculoskeletal Disorders on Full, Partial or Not on Sick Leave.* Journal of Occupational Rehabilitation, 2015. **25**(3): p. 506–517.

26.	Gergele, E., Parent, E. and Gross, EP., *Accuracy of the Orebro Musculoskeletal Pain Questionnaire and Work Assessment Triage Tool for selecting interventions in workers with spinal conditions.* Journal of back and musculoskeletal rehabilitation, 2021. **34**(3): p. 355–362.

27.	Westman, A., et al., *Do psychosocial factors predict disability and health at a 3-year follow-up for patients with non-acute musculoskeletal pain? A validation of the Orebro Musculoskeletal Pain Screening Questionnaire.* European Journal of Pain, 2008. **12**(5): p. 641–649.

28.	Sivan, M.,Sell, B. and Sell, P., *A comparison of functional assessment instruments and work status in chronic back pain.* Eur J Phys Rehabil Med, 2009. **45**(1): p. 31–6.

29.	Boersma, K. and Linton, SJ., *Screening to identify patients at risk: profiles of psychological risk factors for early intervention.* The Clinical journal of pain, 2005. **21**(1): p. 38–43.

30.	Linton, SJ. and Boersma, K *Early identification of patients at risk of developing a persistent back problem: the predictive validity of the Orebro Musculoskeletal Pain Questionnaire.* Clinical Journal of Pain, 2003. **19**(2): p. 80–6

31.	Margison, DA. and French, DJ., *Predicting treatment failure in the subacute injury phase using the Orebro Musculoskeletal Pain Questionnaire: an observational prospective study in a workers' compensation system.* Journal of Occupational & Environmental Medicine, 2007. **49**(1): p. 59–67.

32.	Hockings, RL., McAuley, JH and Maher, CG., *A systematic review of the predictive ability of the Örebro Musculoskeletal Pain Questionnaire.* Spine (03622436), 2008. **33**(15): p. E494–500.

33.	Sattelmayer, M., et al., *Predictive value of the acute low back pain screening questionnaire and the Örebro musculoskeletal pain screening questionnaire for persisting problems.* European spine journal, 2012. **21**(Suppl 6): p. 773–784.

34.	Maher, CG. and Grotle, M., *Evaluation of the predictive validity of the Örebro Musculoskeletal Pain Screening Questionnaire.* The Clinical journal of pain, 2009. **25**(8): p. 666–670.

35.	Dunstan, D.A., et al., *Does the Örebro Musculoskeletal Pain Questionnaire predict outcomes following a work-related compensable injury?* International Journal of Rehabilitation Research, 2005. **28**(4): p. 369–370.

36.	Karran, E.L., et al., *Can screening instruments accurately determine poor outcome risk in adults with recent onset low back pain? A systematic review and meta-analysis.* BMC medicine, 2017. **15**: p. 1–15.

37.	Silva, F.G., et al., *No prognostic model for people with recent-onset low back pain has yet been demonstrated to be suitable for use in clinical practice: a systematic review.* Journal of Physiotherapy, 2022. **68**(2): p. 99–109.

38.	Grotle, M., Vøllestad, NK. and J.I. Brox, JI, *Screening for yellow flags in first-time acute low back pain: reliability and validity of a Norwegian version of the Acute Low Back Pain Screening Questionnaire.* Clin J Pain, 2006. **22**(5): p. 458–67.

39.	Vos, CJ., Verhagen, AP and Koes, BW., *The ability of the Acute Low Back Pain Screening Questionnaire to predict sick leave in patients with acute neck pain.* Journal of manipulative and physiological therapeutics, 2009. **32**(3): p. 178–83.

40.	Hurley, D.A., et al., *How effective is the acute low back pain screening questionnaire for predicting 1-year follow-up in patients with low back pain?* The Clinical journal of pain, 2001. **17**(3): p. 256–263.

41.	Hurley, D.A., et al., *Biopsychosocial screening questionnaire for patients with low back pain: preliminary report of utility in physiotherapy practice in Northern Ireland.* The Clinical journal of pain, 2000. **16**(3): p. 214–228.

42.	Linton, SJ. and Halldén,K., *Can we screen for problematic back pain? A screening questionnaire for predicting outcome in acute and subacute back pain.* Clin J Pain, 1998. **14**(3): p. 209–15

43.	Oxfeldt, M., et al., *Danish short form Örebro Musculoskeletal Pain Screening Questionnaire: Translation, cross-cultural adaptation, and evaluation of measurement properties.* J Back Musculoskelet Rehabil, 2024. **37**(6): p. 1529–1536.

44.	Linton, SJ., et al., *Development of a short form of the Örebro Musculoskeletal Pain Screening Questionnaire.* Spine (03622436), 2011. **36**(22): p. 1891–1895.

45.	Nicholas, M.K., et al., *Predicting Return to Work in a Heterogeneous Sample of Recently Injured Workers Using the Brief OMPSQ-SF.* Journal of occupational rehabilitation, 2019. **29**(2): p. 295–302.

46.	Heikkala, E., et al., *The Örebro Musculoskeletal Pain Screening Questionnaire-Short Form and 2-year follow-up of registered work disability.* European Journal of Public Health, 2023. **33**(3): p. 442–447.

47.	Gabel, CP., et al., *The Örebro Musculoskeletal Screening Questionnaire: validation of a modified primary care musculoskeletal screening tool in an acute work injured population.* Manual therapy, 2012. **17**(6): p. 554–565.

48.	Gabel, CP., et al., *Predictive ability of a modified Örebro Musculoskeletal Pain Questionnaire in an acute/subacute low back pain working population.* European Spine Journal, 2011. **20**(3): p. 449–457.

49.	Gabel, .P., Burkett, B. and Melloh,M., *The shortened Örebro Musculoskeletal Screening Questionnaire: evaluation in a work-injured population.* Manual therapy, 2013. **18**(5): p. 378–385.

50.	Shaw, WS., et al., *The Pain Recovery Inventory of Concerns and Expectations.* Journal of Occupational & Environmental Medicine, 2013. **55**(8): p. 885–894.

51.	Stapelfeldt, C.M., et al., *Cross-cultural adaptation, reliability and validity of the Danish version of the readiness for return to work instrument.* Journal of occupational rehabilitation, 2019. **29**(2): p. 325–335.

52.	Braathen, T., et al., *Psychometric Properties of the Readiness for Return to Work Scale in Inpatient Occupational Rehabilitation in Norway.* Journal of Occupational Rehabilitation, 2013. **23**(3): p. 371–380.

53.	Franche, RL., et al., *The Readiness for Return-To-Work (RRTW) scale: Development and Validation of a Self-report Staging Scale in Lost-time Claimants with Musculoskeletal Disorders.* Journal of Occupational Rehabilitation, 2007. **17**(3): p. 450–72.

54.	Stapelfeldt, CM., *Validation study of the Danish version of the Readiness for Return to Work Scale: Christina Malmose Stapelfeldt.* The European Journal of Public Health, 2017. **27**(suppl_3): p. ckx189. 133.

55.	Shaw, WS., et al., *3rd place, PREMUS best paper competition: development of the return-to-work self-efficacy (RTWSE-19) questionnaire--psychometric properties and predictive validity.* Scandinavian journal of work, environment & health, 2011. **37**(2): p. 109–19.

56.	Momsen, A.-MH, et al., *Cross-cultural adaptation and validation of the Danish version of the 19-item Return-To-Work Self-Efficacy (RTWSE-19) questionnaire.* Scandinavian journal of work, environment & health, 2016. **42**(4): p. 338–345.

57.	Nøttingnes, C., et al., *Jobbrelatert mestringstro ved muskel-og skjelettplager–et spørreskjema.* [Job-related self-efficacy in musculoskeletal disorders - a questionnaire]. Tidsskrift for Den norske legeforening, 2019.139(11).

58.	Button, M., et al., *Return to Work Self-Efficacy (RTWSE-19) Assessment for Patients with Acute Work-Related Low Back Pain.* The Journal for Nurse Practitioners, 2022. **18**(6): p. 660–662.

59.	Razmjou, H., et al., *Measurement properties of the Return-to-Work Self-Efficacy Scale in workers with shoulder injuries.* Physiotherapy Canada, 2019. **71**(1): p. 69–76.

60.	Brouwer, S., et al., *Return-to-work self-efficacy: development and validation of a scale in claimants with musculoskeletal disorders.* Journal of occupational rehabilitation, 2011. **21**(2): p. 244–58.

61.	Brouwer, S., et al., *The predictive validity of the return-to-work self-efficacy scale for return-to-work outcomes in claimants with musculoskeletal disorders.* Journal of occupational rehabilitation, 2015. **25**(4): p. 725–732.

62.	Halpern, M., et al., *The test-retest reliability of a new occupational risk factor questionnaire for outcome studies of low back pain.* Applied Ergonomics, 2001. **32**(1): p. 39–46.

63.	Sternberg, A. and M. Bethge, *Measuring work functioning in individuals with musculoskeletal disorders with reference to the International Classification of Functioning, Disability, and Health: a systematic literature review.* International Journal of Rehabilitation Research, 2018. **41**(2): p. 97–109.

64.	Müller, E., J. Bengel, and M. Wirtz, *Validität und Änderungssensitivität der Skalen zur Erfassung der Funktionsfähigkeit im Beruf (SE-FFB).* Die Rehabilitation, 2014. **53**(03): p. 176–183.

65.	Streibelt, M., *Validity and reliability of a screening instrument for the identification of, extensive work-related problems in patients with chronic diseases (SIMBO-C).* Rehabilitation (Stuttgart), 2009. **48**(3): p. 135–44.

66.	Streibelt, M., et al., *[SIMBO: a Screening Instrument for Identification of Work-Related Disabilities--analyses of construct and prognostic validity].* Rehabilitation, 2007. **46**(5): p. 266–275.

67.	Streibelt, M. and Bethge M, *Prognostic accuracy of the SIMBO regarding future return-to-work problems in patients with mental and musculoskeletal disorders.* Disability and rehabilitation, 2019. **41**(13): p. 1571–1577.

68.	Streibelt, M. and Bethge, M. *Prospective Cohort Analysis of the Predictive Validity of a Screening Instrument for Severe Restrictions of Work Ability in Patients with Musculoskeletal Disorders.* American Journal of Physical Medicine & Rehabilitation, 2015. **4**(8): p. 617–626.

69.	Mittag, O., et al., *[Predictive validity of a brief scale to assess subjective prognosis of work capacity (SPE Scale) in a cohort of LVA insured patients with severe back pain or functional complaints relating to internal medicine].* Soz Praventivmed, 2003. **48**(6): p. 361–9.

70.	Fauser, D., et al., *Subjektive Erwerbsprognose als Indikator für Rehabilitationsbedarf: eine Kohortenstudie bei Versicherten mit Rückenschmerzen.* [*Self-Reported Prognosis of Employability as an Indicator of Need for Rehabilitation: A Cohort Study in People with Back Pain*]*.* Rehabilitation, 2022. **61**(2): p. 88–96.

71.	Stienstra, M., et al., *Measurement Properties of the Work Ability Score in Sick-Listed Workers with Chronic Musculoskeletal Pain.* Journal of Occupational Rehabilitation, 2022. **32**(1): p. 103–113.

72.	Boekel, I., et al., *Validation of the Work Ability Index-single item and the pain disability index-work item in patients with chronic low back pain.* European spine journal : official publication of the European Spine Society, the European Spinal Deformity Society, and the European Section of the Cervical Spine Research Society, 2022. **31**(4): p. 943–952.

73.	Boekel, I., et al., *Pain Disability Index–Work item in patients with chronic low back pain.* Groningen Spine Cohort, 2022. **31**(4): p. 241.

74.	Fauser, D., et al., *Work Ability Score as predictor of rehabilitation, disability pensions and death? A German cohort study among employees with back pain.* Work, 2022. **73**(2): p. 719–728.

75.	Zetterberg, H., et al., *Psychometric Assessment of the Swedish version of the Work Ability Index in Patients with Chronic Pain in Specialized Care.* J Rehabil Med, 2023. **55**: p. jrm7146.

76.	Russo, F., et al., *Importance of an Integrated Assessment of Functional Disability and Work Ability in Workers Affected by Low Back Pain.* Safety and Health at Work, 2024. **15**(1): p. 66–72.

77.	Kujala, V., et al., *Classification of Work Ability Index among young employees.* Occupational medicine (Oxford, England), 2005. **55**(5): p. 399–401.

78.	Rashid, M., et al., *Do work ability and life satisfaction matter for return to work? Predictive ability of the Work Ability Index and life satisfaction questionnaire among women with long-term musculoskeletal pain.* BMC Public Health, 2021. **21**(1): p. 1–9.

79.	Kuijer, PPFM., et al., *Prognostic value of self-reported work ability and performance-based lifting tests for sustainable return to work among construction workers.* Scandinavian Journal of Work, Environment and Health, 2012. **38**(6): p. 600–603.

80.	Makishima, M., et al., *Validity and responsiveness of the Work Functioning impairment scale (WFun) in workers with pain due to musculoskeletal disorders.* Journal of occupational health, 2018. **60**(2): p. 156–162.

81.	Roy, J.-S., Desmeules, F., and MacDermid, J., *Psychometric properties of presenteeism scales for musculoskeletal disorders: a systematic review.* Journal of rehabilitation medicine, 2011. **43**(1): p. 23–31

82.	Tang, K., et al., *Confirmatory Factor Analysis of the Work Limitations Questionnaire (WLQ-25) in Workers' Compensation Claimants with Chronic Upper-Limb Disorders.* Journal of Occupational Rehabilitation, 2013. **23**(2): p. 228–238.

83.	Lu, Z., et al., *An Evaluation of the Structural Validity of the Work Limitation Questionnaire Using the Rasch Model.* Archives of Physical Medicine and Rehabilitation, 2021. **102**(4): p. 633–644.

84.	Roy, J.-S., et al., *Validity and Responsiveness of Presenteeism Scales in Chronic Work- Related Upper-Extremity Disorders.* Physical Therapy, 2011. **91**(2): p. 254–266.

85.	Tang, K., et al., *Comparison of the psychometric properties of four at-work disability measures in workers with shoulder or elbow disorders.* Journal of Occupational Rehabilitation, 2009. **19**(2): p. 142–54.

86.	Beaton, DE. and Kennedy, CA., *Beyond return to work: testing a measure of at-work disability in workers with musculoskeletal pain.* Quality of Life Research, 2005. **14**(8): p. 1869–79.

87.	Thygesen, K.B., et al., *Translation and cross-cultural adaptation of the Work Rehabilitation Questionnaire (WORQ) into Danish.* Frontiers in Rehabilitation Sciences, 2023. **4**: p. 1134039.

88.	Hansen, A., et al., *Reliability and Construct Validity of the Work Rehabilitation Questionnaire Domains in Patients with Persistent Low Back Pain.* J Occup Rehabil. 2024 Nov 8.

89.	Hansen, A., et al., *Assessing Work Functioning in Patients with Persistent Low Back Pain: Exploring the Structural Validity of the Work Rehabilitation Questionnaire.* Journal of Occupational Rehabilitation, 2024. **34**(3): p. 669–682.

90.	Aerts, BRJ., et al., *Development of a novel WOrk-Related Questionnaire for UPper extremity disorders (WORQ-UP).* International Archives of Occupational and Environmental Health, 2017. **90**(8): p. 823–833.

91.	Aerts, BR., et al., *WOrk-Related Questionnaire for UPper extremity disorders (WORQ-UP): factor analysis and internal consistency.* Archives of physical medicine and rehabilitation, 2018. **99**(9): p. 1818–1826.

92.	Johansen, T., et al., *Cross-cultural adaptation of the Work Role Functioning Questionnaire 2.0 to Norwegian and Danish.* Work (Reading, Mass.), 2018. **59**(4): p. 471–478.

93.	Goorts, K., et al., *Quickscan Assesses Risk of Long-Term Sickness Absence: A Cross-Sectional Validation Study.* Journal of Occupational and Environmental Medicine, 2019. **61**(2): p. E43–E50.

94.	Goorts, K., et al., *Screening for the risk on long-term sickness absence.* J Public Health Res, 2018. **7**(2): p. 1419.

95.	Du Bois, M., et al., *A screening questionnaire to predict no return to work within 3 months for low back pain claimants.* European Spine Journal, 2008. **17**(3): p. 380–385.
